# Delphi Technique on Nursing Competence Studies: A Scoping Review

**DOI:** 10.3390/healthcare12171757

**Published:** 2024-09-03

**Authors:** Luís Furtado, Fábio Coelho, Sara Pina, Cátia Ganito, Beatriz Araújo, Cândida Ferrito

**Affiliations:** 1 Faculty of Health Sciences and Nursing, Universidade Católica Portuguesa, 1649-023 Lisboa, Portugal; s-sfmpina@ucp.pt (S.P.); s-cganito@ucp.pt (C.G.); baraujo@ucp.pt (B.A.); candida.ferrito@ucp.pt (C.F.); 2 Department of Nursing, Mental Health and Gerontology, School of Health, University of the Azores, 9700-042 Angra do Heroísmo, Portugal; fabio.ad.coelho@uac.pt; 3 Center for Interdisciplinary Research in Health, Faculty of Health Sciences and Nursing, Universidade Católica Portuguesa, 4169-005 Porto, Portugal; 4 Center for Interdisciplinary Research in Health, Universidade Católica Portuguesa, 1649-023 Lisboa, Portugal

**Keywords:** Delphi technique, consensus, nursing, review, professional competence, methodological discussion, expert survey

## Abstract

This scoping review was conducted under the Joanna Briggs Institute (JBI) framework. It included primary studies published until 30 April 2023, obtained through a systematic search across PubMed, Web of Science, CINAHL, and MEDLINE databases. The review focused on primary studies that used the Delphi technique in nursing competence research, especially those related to defining core competency frameworks and developing instruments to assess professional competence. The goal was to analyze the different methodological approaches used by authors, synthesize them, and propose recommendations to enhance methodological rigor, reliability, and validity in the application of the Delphi technique. For this purpose, the following review question was established: “What is the available evidence on the use of the Delphi technique in the study of professional competence in nursing?”. The extracted textual elements underwent a content analysis, resulting in dimensions established through an inductive approach. Twenty studies were included, yielding insights into diverse methodological options for conducting Delphi studies, organised around a set of dimensions: (1) preparatory procedures; (2) procedures for accessing and selecting experts; (3) acquisition of expert input; (4) data analysis and consensus; and (5) ethical and legal procedures and guarantees. The study’s limitations include the inability to include certain studies due to a lack of response to requests for clarification from corresponding authors. Additionally, the primary studies’ methodological quality was not assessed, which is another relevant aspect. The study’s results offer valuable insights for researchers intending to utilise the Delphi technique within the context of the research referenced in the included studies. This information encompasses important methodological choices, highlighting their potential benefits and associated risks. The review was prospectively registered on the Open Science Framework (Registration No: osf.io/kp2vw).

## 1. Introduction

Professional competence is a dynamic and complex concept that is constantly being constructed. It translates the knowledge of how to do things under the influence of working relationships, organisational culture and contingencies inherent to professional practice contexts [1]. In nursing, the understanding of competence is strongly influenced by Patricia Benner’s perspective, for whom competence was about performing a task with desirable results under real circumstances, in which nurses progressed from “beginners” to “experts” through experience and time [2]. In other words, professional nursing competence corresponds to the ability of nurses, in different professional practice contexts, to combine complex attributes of care practice, including knowledge, skills, values and attitudes [3], as holistic units that are understood in the context of clinical practice, necessary to performance effectively in the nursing environment [4].

The definition of core competency frameworks in different areas of nursing or the development of instruments capable of evaluating nurses’ professional competence involves a set of particularly demanding and complex methodological procedures, which often use the Delphi technique in their designs [5,6,7,8].

The next subsection of the document provides a theoretical explanation of the main aspects related to the processes inherent to the application of the Delphi technique.

### 1.1. The Delphi Technique

The Delphi technique is a significant method for achieving consensus across various disciplines, such as education, psychology, sociology, management, and healthcare, including nursing [9,10,11]. In the health sciences, it identifies research priorities and develops clinical guidelines [12,13,14,15,16]. Selecting a consensus method, like Delphi, depends on the study’s objectives, existing scientific evidence, participant interaction models, available time, and costs [13]. Delphi is ideal when empirical evidence is limited or unclear [17,18,19] when anonymity is needed, and when gathering geographically dispersed participants is impractical [13,14,20,21]. The technique relies on the idea that “two heads are better than one” especially when face-to-face interaction might lead to conformity pressures [13,15,20,22,23].

Delphi studies address biases in group discussions by adhering to three key principles [10,13,15,18,21,22,24,25,26,27,28,29]: (1) anonymity, where participants’ identities and responses are kept confidential; (2) iteration with controlled feedback, allowing participants to refine their insights through multiple rounds of summarised feedback; and (3) statistical group responses, synthesising individual opinions into an objective analysis at each round’s conclusion. However, complete anonymity cannot be guaranteed, as researchers know the panellists’ identities, and panellists may know each other and share opinions, leading to the concept of “quasi-anonymity” [9,29,30,31].

There are various Delphi study types, including classic, modified, decision-making, and real-time Delphi [32,33]. The choice of type depends on the research problem and objectives as well as participant and utilisation requirements. Classic Delphi studies typically involve two or more rounds of questionnaires. Initially, experts provide opinions through open questions, which are analysed and returned as statements or closed questions for further rounds. This continues until a consensus is reached [14,18,27]. In Modified Delphi, the first round may involve face-to-face interviews or literature-based statements instead of open questions [27,34].

In Delphi studies, researchers serve as “planners” and “facilitators”, ensuring minimal bias by maintaining objectivity and providing controlled feedback. Regardless of their agreement, they must avoid influencing outcomes, focusing solely on supplying impartial and relevant information to the expert panel [12,21,25].

#### 1.1.1. Selection and Composition of the Expert’s Panel

The expert panel selection is the most critical stage in a Delphi study, as it significantly impacts the quality of the results [9,10,21,35]. Unfortunately, no standardised objective criteria for selecting participants exists [11,27,36,37]. Eligibility for a Delphi study generally depends on several factors: relevant background and experience in the subject matter, ability to provide valuable insights, willingness to revise judgments to achieve consensus, sufficient time to participate, enthusiasm for the topic, and good communication skills [9,21,31,38]. It is also essential to consider individuals who may use the study’s results or find it meaningful [21,29,37]. However, even though all participants are referred to as “experts”, their levels of expertise can vary depending on their specific backgrounds [13,21,37].

A careful evaluation of the qualifications of the Delphi panellists is crucial [9]. The most appropriate individuals are typically identified through a nomination process, with recommendations from key figures in the field, authors of notable publications, or individuals with access to privileged information related to the study [9,36]. Establishing objective criteria, such as a minimum number of publications, specific certifications, years of professional experience, or academic qualifications, can also be helpful [17,31,39,40].

The number of experts included in the panel is another vital consideration [31]. No methodological guideline exists on the ideal number of panellists [9,12,27,41]. Researchers should aim to include the minimum number of participants required to represent a range of relevant opinions, ensuring that this number aligns with the research team’s capacity to process the data [17,29,38,41]. A panel that is too large can result in lower response rates, raise concerns about the representativeness of the results, and complicate time management [21,42].

Despite careful selection, a Delphi study may still be vulnerable to participant-related biases. Common biases include desirability bias, groupthink (bandwagon effect), and anchoring bias, where participants’ responses are overly influenced by their existing knowledge [43]. Researchers need to be mindful of these biases and take steps to mitigate their impact on the study.

#### 1.1.2. Rounds

The Delphi process is repeated until consensus is reached, typically within three or four rounds, although no optimal number has been established [9,13,21]. In a classic Delphi study, the first round usually involves distributing a questionnaire with open-ended questions designed to generate ideas and explore perspectives on the topic [9,10,29]. After collecting responses, the researchers transform the data into statements, forming the basis of a structured questionnaire for the second round [29]. Alternatively, a structured questionnaire based on literature reviews, containing statements instead of open-ended questions, may be used from the first round [18,21].

In the second round, each Delphi participant receives a questionnaire to review the items or statements compiled by the researchers based on the first round’s data and expert feedback [9,42]. This questionnaire is derived from the questionnaire sent to the experts in the first round, after analysing the data, recommendations and observations provided by the experts. Participants rate each item’s importance, relevance, clarity, and appropriateness or prioritise them, highlighting areas of agreement and disagreement (consensus) [18,21]. Responses are typically collected via a Likert scale or free-text box for suggestions.

In the third round, participants receive a summary of the items and ratings from the previous round. They are asked to revise their judgements or explain their choices [10,12,16,29]. This process refines the panel’s insights and improves consensus [18,21].

As the Delphi method reaches the fourth and often final round, participants are presented with the remaining items, their rankings, minority opinions, and consensus items. They are given a final chance to revise their judgements. The number of Delphi iterations, including this final round, depends on the level of consensus required and may extend beyond four rounds if needed [18,21,42].

#### 1.1.3. Data Analysis and Consensus

Data can be analysed using qualitative and quantitative approaches, providing researchers with diverse options [9,42,44]. Qualitative data processing is essential in classic Delphi studies (which use open-ended questions in the first round), modified Delphi studies (where group dynamics generate data), and subsequent rounds that gather qualitative feedback [17,44]. Quantitative analysis typically involves central tendency measures (mean, median, mode) and dispersion measures (standard deviation, interquartile range) [14,17,21,45].

Consensus is a key objective in most Delphi studies, and its criteria should be defined before the expert panel begins [10,27,38]. However, consensus does not imply complete agreement or that the correct answer has been found [12,29,31]. Instead, it is a pre-established level of agreement, typically measured using a Likert-type scale, with an average score determined in advance for each statement [9,13,27].

Consensus can be achieved by meeting a predetermined percentage threshold, assessing response stability across rounds, or aggregating judgments [17,27,46,47]. The most common criterion is based on the percentage of agreement or the percentage of participants who rate statements at the extremes of the Likert scale, indicating acceptance or rejection [11,45,48].

#### 1.1.4. Reliability and Validity

The ultimate goal of any researcher is to produce valid, reliable, sensitive, impartial, and comprehensive results [49,50]. Rigour is crucial to ensuring reliability in Delphi studies. However, this has been challenging due to ongoing modifications to the method and the emphasis on reliability and validity in quantitative research [9,33].

To ensure reliability, researchers must apply transparent methodological procedures that enhance credibility and minimise errors and biases. This ensures that results remain consistent if the study is replicated. Similarly, enhancing validity requires selecting a panel of experts based on objective criteria, ensuring they have genuine expertise, are impacted by the results, and can represent a diverse range of perspectives on the topic [41].

#### 1.1.5. Advantages and Disadvantages of the Delphi Technique

The Delphi technique offers several advantages, such as incorporating diverse opinions from geographically dispersed participants and promoting inclusivity [11,29]. It is also cost-effective, requiring minimal support infrastructure. Additionally, the technique reduces the influence of dominant individuals through anonymity and minimises the distractions of group settings [10,27,36,41]. By consulting experts, the method generates valuable insights and contributes to solving complex problems. The iterative rounds allow participants to reflect deeply, positively affecting the validity of the results [18].

However, the Delphi technique also has drawbacks [21]. The iterative process can lead to participant fatigue, causing low response rates, compromising subsequent rounds and the representation of diverse opinions [11,13,21,27,31]. It can also be time-consuming, as researchers must wait for responses before analysing and processing data for feedback in the next round [9,12,13,21].

### 1.2. Rationale, Context and Aim of the Scoping Review

Notwithstanding this, the Delphi technique has been criticised for various shortcomings, particularly in terms of the systematisation, detail and justification of the methodological procedures defined by the researchers who use the technique, leading to great variability in its use, but also in terms of the reporting of studies that refer to its use [21,27].

Following the significant publications of Keeney [9,29] regarding the use of the Delphi technique in 2006 and 2011, respectively, and in response to the lack of a comprehensive literature synthesis analysing the methodological procedures employed in studies on the topic of nurses’ professional competence using the Delphi technique, the authors, as part of their effort to design a robust Delphi study capable of addressing the common criticisms associated with the use of this method, aimed to analyse the different methodological approaches used among other authors, synthesise them, and propose recommendations to enhance methodological rigour, reliability and validity in the Delphi technique application, focused on identifying preparatory procedures, expert selection procedures and contact methods, data collection and analysis steps, and the consensus-reaching process. The Delphi study that the authors will develop following this scoping review is part of a more comprehensive research project focussing on creating a new instrument for assessing nurses’ professional competence in evidence-based practice.

In the process, the research team discussed whether to opt for a systematic or scoping review, namely, which option would allow the objective to be achieved. While a systematic review seeks to assess the feasibility, suitability, significance or effectiveness of a particular treatment or practice, a scoping review is more suitable for studies focused on identifying characteristics or concepts in articles or research, as well as mapping, reporting or discussing these elements [51]. In this case, a scoping review was developed, as it was considered more appropriate, allowing us to outline the set of issues, approaches, interests and opportunities within a specific area.

Therefore, this scoping review aimed to explore, analyse, and synthesise the diverse methodological approaches employed in primary studies using the Delphi technique to assess professional competence in nursing. This review seeks to formulate recommendations addressing common criticisms of the Delphi technique, enhancing its methodological robustness and reliability in future research.

## 2. Materials and Methods

The scoping review used the JBI methodology for scoping reviews [50,51,52], and the review process (study design, database searches, screening and selection of studies, extraction, synthesis and report production) occurred between January 2023 and April 2024 and involved the following steps [50,51,52]: (a) formulating the search question; (b) identifying relevant sources of evidence; (c) selecting sources of evidence for inclusion; (d) collecting and extracting data; and (e) organising, summarising, and presenting the findings.

The results of the search in scientific literature databases, including the process of screening and selecting documents, were presented using a process flowchart following the Preferred Reporting Items for Systematic Reviews and Meta-Analyses Extension for Scoping Reviews (PRISMA-ScR) guidelines (Supplementary Materials Tables S1 and S2) [53,54]. The review protocol was registered on the Open Science Framework platform [55]. The review question “What is the available evidence on the use of the Delphi technique in the study of professional competence in nursing?” was established as the guiding principle for the review study and structured according to the PCC mnemonic (“participants”, “concept” and “context”).

This literature review stemmed from a series of methodological procedures conducted as part of an ongoing research project to develop an instrument to assess nurses’ competence in evidence-based practice. The forthcoming instrument’s development will utilise the Delphi technique to validate the content of the respective statements. Before conceiving the Delphi study, the authors opted to conduct a comprehensive review of studies conducted in this field of research employing the Delphi technique. This approach was chosen to enhance the reliability and validity of the results and overcome some of the criticisms levelled at the technique.

The literature review protocol was registered in the Open Science Framework platform with the DOI: 10.17605/OSF.IO/KP2VW [55].

### 2.1. Eligibility Criteria

Following the JBI framework for scoping reviews of the literature [56], the team of reviewers collaboratively defined a set of inclusion and exclusion criteria, against which they subjected the records obtained from the searches conducted, as detailed below:

- Participants. This review considered studies whose participants were general care nurses, specialist nurses, and advanced practice nurses (level of nursing practice that utilizes extended and expanded skills, experience, and knowledge in assessment, diagnosis, treatment, and care management, often holding advanced degrees and capable of independent practice [57]). Studies in which the participants were undergraduate nursing students, university professors or educators in higher education settings, and auxiliary nursing staff were excluded. In studies with more than one healthcare professional group, when this was clear, only the dimension of the results concerning nurses was considered.
- Concept. The literature review considered studies that explicitly used the Delphi technique.
- Context. The review encompassed studies on content validity regarding the developing instruments for measuring and evaluating nursing competence and those defining core competency frameworks for nurses’ professional competence across the diverse areas that characterise professional nursing practice.
- Types of sources. Only primary studies were included independently or as part of more extensive studies. Likewise, only studies with explicit information on the different stages and processes underlying the use of the Delphi technique were included. The review team’s decision to restrict the scoping review to primary studies was due to the need to collect all the methodological elements related to the use of the Delphi technique directly from the source.

### 2.2. Search Strategy

A preliminary exploration was undertaken across several platforms, including PROSPERO, Open Science Framework, Cochrane Database of Systematic Reviews, and JBI Evidence Synthesis, with no recent or ongoing literature reviews being found addressing the specific topic and purpose of this literature review. Subsequently, a three-step search strategy was employed to identify primary studies published up to 30 April 2023, meeting the established inclusion and exclusion criteria. Language restrictions were not applied, and documents in languages other than English and Portuguese (the authors’ mother language) were assessed based on their titles and abstracts in English. Those meeting preliminary inclusion criteria underwent professional translation for full-text analysis.

The initial step involved a limited search to identify relevant studies, followed by analysis of text, words, titles, abstracts of the identified articles, and indexed terms and keywords. These words were used to formulate an initial search strategy in the MEDLINE database (via EBSCO) to ascertain the evidence available for the literature review. Subsequently, an extended search (step two) on PubMed, Web of Science (Clarivate), CINAHL (via EBSCO), and MEDLINE (via EBSCO) databases, employing the defined search strategy (see Table 1). Finally, in the third step, the reference lists of all documents selected for inclusion were manually checked, and other relevant documents were identified. Table 1 illustrates the search strategy used in MEDLINE (via EBSCO), further adapted to the other databases’ specificities (see Supplementary Materials Table S3).

### 2.3. Study Selection

**Figure 1:**
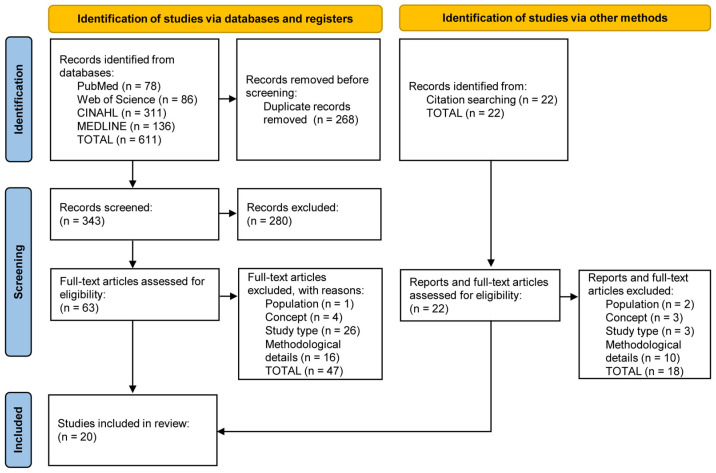
PRISMA-ScR flowchart relating to identifying, screening and selecting the documents included in the literature review.

The acquired records were exported and transferred to EndNote^®^ v.20.4 software (Clarivate Analytics, Philadelphia, PA, USA) for organisation, analysis, and initial removal of duplicates. The analysis, categorisation, and selection process occurred in two phases, performed on the Rayyan^®^ platform (https://www.rayyan.ai/, Qatar Computing Research Institute, Doha, Qatar). Initially, the records were imported into the Rayyan^®^ platform, where they underwent a secondary check for duplicates, followed by screening based on title and abstract, adhering to the established eligibility criteria, by two independent reviewers blinded to each other’s assessments. In a subsequent phase, the studies deemed suitable for full-text analysis were re-imported into Rayyan^®^ and re-evaluated against the established eligibility criteria by two independent reviewers, employing a blinded approach. The reasons for exclusion were documented and reported. Any discrepancies between reviewers were resolved through discussion or, if necessary, with the involvement of a third reviewer. Given the review’s nature and objectives, the review team opted not to evaluate the methodological quality of the included studies, a circumstance that was recognised and further elaborated in the study’s limitations statement [58]. Contact was made with the corresponding authors whenever the information provided in the paper was considered insufficient; in cases where the corresponding author did not respond, and the sought information was vital for the reliability of the extracted data, the study was excluded. The flowchart in Figure 1 illustrates the total number of identified records, the included and excluded reports, the reasons for exclusion, and documents incorporated following manual scrutiny of the reference lists.

### 2.4. Data Extraction and Presentation

The authors utilised a specifically designed tool within Microsoft Excel^®^ 365, version 2406 (Build 17726.20126) to extract data, which was tested on a random selection of 10 documents to ensure clarity and effectiveness in extracting relevant information for the study [52]. No adjustments were deemed necessary after analysing and discussing the results obtained from testing the extraction tool. For each study included, the following data points were extracted: publication year, authors, journal name, title, country, the study aims, preparatory procedures, expert access procedures, expert selection procedures, instrumentation and data analysis. Data extraction was performed independently by two reviewers. The data from the included studies were presented using a descriptive narrative supported by tables.

## 3. Results

After screening and selecting the 611 records extracted from scientific databases (PubMed—78; Web of Science—86; CINAHL—311; and MEDLINE—136) and the 22 documents obtained from other sources. After the screening and selection process, a total of 20 original primary studies were included in the literature review (Figure 1).

The chronological distribution of the included studies spans from 2005 to 2022, where 60% were published in the last 5 years [59,60,61,62,63,64] and 75% in the last 10 years [59,60,61,62,63,64,65,66,67,68,69,70]. The three most representative years in the sample were 2022 [63,64,71], 2021 [60,61,62] and 2019 [68,69,70], with three studies each. The included studies were conducted across 11 countries, with China being the most represented among them, contributing seven studies [60,62,64,66,67,68,71]. Regarding the scope of the Delphi technique’s application, twelve of the included documents utilised this approach on the content validity of instruments to assess nurses’ professional competence, while eight documents employed the technique to establish core competency frameworks in nursing. Most studies used a modified Delphi procedure, and the authors’ methodological choices varied considerably between studies. In addition, some reports did not consistently provide all the methodological details regarding their full options. Please refer to Supplementary Materials Table S4 for a comprehensive summary of the elements extracted from each document in the scoping review.

The results will be presented and organised around five main dimensions, which emerged from an inductive process based on the data obtained during the exhaustive analysis of the studies included in the literature review. This approach does not start from a predefined theoretical structure and reflects the emerging findings of this literature review study. These dimensions were thus defined because they represent the stages and processes deemed significant for operationalising the Delphi technique in studying nurses’ professional competence. This approach was chosen because it offers a comprehensible framework for presenting all the gathered information. The five main dimensions are as follows: (1) preparatory procedures; (2) procedures for accessing and selecting experts; (3) acquisition of expert input; (4) data analysis and consensus; and (5) ethical and legal procedures and guarantees. Table 2 shows the stages, processes and methodological options used in the Delphi studies included in the literature review.

### 3.1. Preparatory Procedures

In some studies, researchers undertook literature reviews and theoretical analyses [60,61,62,64,67,68,72,73,74], which incorporated elements from competency frameworks documented by regulatory bodies overseeing nursing practice and by international professional nursing organisations [62,63,64,68]. Concomitantly, other studies employed a combination of literature reviews and expert consultation techniques to organise and synthesise information [59,61,67,71]. These techniques included using focus groups [61,63,71] and other discussion group methods [59,60,66,71,73]. Some authors established the initial step of their studies to pose open-ended questions to experts other than the panellists who made up the Delphi panel instead of performing literature reviews [65,70,76,77]. Some studies mentioned employing discussion procedures within the research team to ensure the consistency of the information obtained [66,67,71,75].

### 3.2. Access and Expert Selection Procedures

The average number of experts per panel was 43, ranging from 7 [61] to 181 [78]. Experts were recruited via direct contact by the research team [60,63,77] or referrals from the experts themselves [59]. The snowball method was also used, where newly recruited experts invited others [59,65,67]. Experts were identified on relevant organisations’ online platforms [59,75], or organisations were directly engaged in nominating personnel based on predefined criteria [63,71,73]. Recruitment also included electronic newsletters, institutional platforms, and participation in scientific meetings [69,75]. Researchers reviewed relevant articles to identify authors within a set timeframe [77], often employing multiple strategies to recruit experts.

The experts selected for the Delphi panels in the reviewed studies were primarily chosen to represent diverse facets, sensitivities, and understandings of the studied topics [69,72,76]. Selection criteria included their specific roles, such as nursing professors, educators, researchers, nurse managers, specialists and general care nurses, and those from various clinical settings with different levels of training and academic degrees, as well as professionals from regulatory bodies were also included [59,60,61,64,65,66,67,69,70,71,73,74,76,78,79], reflecting geographical and cultural diversity where possible [59,67,77]. Additionally, professional experience, typically a minimum of 5 to 10 years, was a common criterion [60,62,63,64,66,67,68,69,71,72,73,77]. Some studies also required availability for all Delphi phases [60,67,70].

Experts were primarily invited to participate in the study via email, which outlined the study’s aims and procedures [59,65,69,70,71,73,75]. Additional methods included telephone calls and face-to-face interactions facilitated by the researchers [69]. In some cases, a research team member embedded within the experts’ professional environments acted as an intermediary [72].

All reviewed studies retained the same expert panel through successive rounds unless experts chose to withdraw, with one exception [61]. In this case, the authors selected the final panel based on an absence of “atypical” assessments in the first round, defined by extreme scores and a qualitative evaluation of feedback.

### 3.3. Acquisition of Experts’ Inputs

Similar to determining the appropriate number of experts for a Delphi panel, no formal recommendation exists for the maximum number of rounds needed to achieve consensus. In the studies reviewed, consensus was typically reached by the second round [61,62,63,64,66,68,71,72,74], with four rounds being the maximum observed [69,70,73,77].

#### 3.3.1. Instrumentation

The questionnaire was the primary data collection method [59,61,69,70,72,73,74,76], usually distributed via email, in digital format, or through an online platform [38]. Some studies enhanced questionnaires with semi-structured interviews conducted by telephone, face-to-face, or in group settings, initially using an open-ended approach before shifting to closed-ended questions in later rounds [60,63,65,66]. Only two studies used postal questionnaires [77,78].

In terms of structure, studies [59,60,69,72,75] commonly organised instruments into three sections: (i) instructions and informed consent (initial round); (ii) panellist characterisation (initial round); and (iii) the main questionnaire, with its format and content varying by round.

The validation content aimed at achieving expert consensus was assessed using Likert-type scales to evaluate relevance, specificity, and comprehensibility. Experts could justify their ratings, offer suggestions, and ask questions. Most studies used 5-point scales (e.g., 1—“unimportant” to 5—“very important”; 0—“not competent at all” to 4—“very competent”) [59,60,63,65,66,68,69,70,72,73,76,78], though some used 4-point (e.g., 1—“not important” to 5—“very important”) [61,62], 6-point (e.g., 1—“strongly disagree” to 5—“strongly agree”) [75], and 7-point scales (e.g., 1—“not at all important” to 7—“very important”) [77].

Regarding the time allotted for experts to respond, the studies referencing this information mentioned periods ranging from seven days [69], ten days [70], fourteen days [66], and twenty-eight days [63], with many studies omitting this detail altogether.

#### 3.3.2. First Round

Some studies began by assessing the relevance, specificity, and comprehensibility of validated content using closed-ended questionnaires with Likert-type scales. These studies also included free-text fields for experts to provide feedback, ask questions, and suggest improvements [59,61,69,73,75,76]. In contrast, other authors used less restrictive methods for the initial round of Delphi studies, employing interviews [65,66,77], group meetings [60,63], or open-ended questions [70,72,78]. The data from this exploratory phase underwent content analysis, involving classification and categorisation based on theoretical structures [70]. After anonymising, the compiled information, including unresolved statements, was returned to experts for further review and feedback, incorporating suggestions and clarifications [59,60,65,69,71,73,75,76].

#### 3.3.3. Subsequent Rounds

In the following rounds, the goal was to reach consensus among experts on statements unresolved in the initial round and on information from the content analysis of responses to open-ended questions, interviews, and group discussions. This information was then organised into domains, subdomains, competence criteria, and items [59,61,63,65,71,72].

In these rounds, a more structured approach is often used, with Likert-type scales enabling experts to rate various elements [60,61,65,66,68,69,70,73,76,78]. Although the questions are objective, some studies permit experts to express opinions, raise doubts, and suggest changes throughout rounds [70,75]. This may include introducing new elements not considered initially [70]. However, as the study advances and consensus is sought, the opportunity for expert input tends to diminish, with researchers often using closed-question questionnaires [65,70].

The results were anonymised and shared with experts from the previous round in each subsequent round. This process allowed experts to monitor the panel’s collective evolution and reassess their positions based on the feedback and additional information provided by the researchers [60,65,73,75,76,77].

#### 3.3.4. Stability of the Expert Panel

In the studies reviewed, the average attrition rate was 16.6%, ranging from no dropouts [60,62,66,70,71,72,74] to a peak of 69.1% [78]. Besides the study with the highest attrition, several others showed significant attrition rates, ranging from 34.4% to 61.9% [59,69,73,75,77].

### 3.4. Data Analysis and Consensus

Data from the Delphi rounds were processed according to their nature, using either interpretative analysis or statistical and consensus measures. Content analysis was applied to data from group dynamics, interviews, open-ended questions, and expert comments, including categories, thematic areas (domains and subdomains), and statements. Different researchers carried out this process in each study to ensure all potential domains, subdomains, and items were thoroughly identified [65,69,72,77].

Data on experts’ evaluations and their characteristics were subjected to statistical treatment for questionnaire analysis. These data were presented using absolute and relative frequencies, as well as measures like the mean and standard deviation [60,65,70,73,78].

Only three studies explicitly stated that they established consensus among experts before starting the study [72,73,76]. In one case, the authors adjusted the initial consensus level to avoid extensive content validation in the first round. They justified this modification by emphasising the dynamic and interactive nature of the Delphi technique and the need to encourage in-depth discussions on the topic [65].

Most studies in the literature review used the Content Validity Index (CVI) [61,62,63,66,67,68,69,70,72,73,74,76], with a predetermined cut-off point to assess the relevance of items or statements. Items were retained or removed based on the proportion of experts who found them relevant. CVI values ranged from 0.75 to 0.90 [61,62,63,65,67,69,76]. Additionally, some studies used the mean and standard deviation to evaluate opinion direction and expert agreement [63,70,73,75,78]. Measures such as Kendall’s W (expert coordination coefficient) and the coefficient of variation (CV) were also employed [60,64,66,67,71], along with the weighted Kappa coefficient (Kw), to assess expert coordination and consensus [67].

### 3.5. Ethical–Legal Procedures and Guarantees

Of the studies reviewed, only 13 explicitly detailed their ethical and legal procedures, ensuring confidentiality and anonymisation of information before sharing it with experts in successive Delphi rounds [59,61,62,64,69,70,71,72,73,74,76]. Some studies, however, could not maintain complete anonymity during initial rounds due to group dynamics, where experts knew each other’s identities. Despite this, the confidentiality of the information from group dynamics was upheld, and anonymity was maintained for questionnaire data in subsequent rounds [60,63]. Only four studies explicitly mentioned obtaining informed consent from experts, conducted in the initial round [60,69,71,73].

None of the included studies explicitly mentioned safeguards for protecting collected data, particularly regarding compliance with legal requirements. Depending on the country of the study, such safeguards may be essential, especially when data are collected via digital platforms. Compliance with data protection laws can vary widely by jurisdiction. It may involve obtaining informed consent, data encryption, secure storage protocols, and adherence to the General Data Protection Regulation [80,81] in the EU or the US Health Insurance Portability and Accountability Act [82]. Although these procedures were not detailed in the reviewed studies, researchers should consider and follow relevant legal requirements to ensure data privacy and confidentiality.

## 4. Discussion

Among the included studies, different approaches were used to formulate evaluation elements for expert panels, including theoretical and literature reviews, focus groups, expert consultations, and internal team discussions. The most robust approach for studying nurses’ professional competence is to use a theoretical and literature review followed by analysing and categorising the collected information, since it offers a solid base of pre-existing knowledge, identifying trends, gaps and established practices in the area. This approach also facilitates the integration of consolidated theories and models, contributing, for example, to constructing more robust and evidence-based competence profiles. This method ensures that the evidence presented to the expert panel is based on scientific research [9,13,17,29,36,83], rather than solely on expert opinion. This systematisation may fall short when the available evidence is ambiguous or insufficient [17].

Some studies have involved presenting literature review results to external experts for analysis beyond the Delphi study. This approach carries risks, such as potential reliability issues, including the omission of key elements or the inclusion of irrelevant ones. Ideally, the research team should organise the literature review [17,29,83]. The expert panel in the Delphi study should handle initial analysis using an exploratory approach that does not seek consensus immediately, ensuring that a well-chosen panel evaluates all elements. Additionally, Delphi studies are time-consuming; adding extra stages beyond the standard design can extend the study duration without improving results [29,83].

Expert panel composition is crucial in Delphi studies [29,35]. Researchers should assemble a diverse group of experts [17,37] to capture a wide range of opinions, understandings, and perspectives. This approach enhances the validity of the findings by incorporating varied judgments on the topic under investigation [10,25,29,35,37,38,41,79,83,84]. This is particularly relevant when it comes to studying professional competence, firstly and foremost, because researchers must endeavour to capture all the existing perspectives on a given area of competence; otherwise, the panel that is set up in the meantime will be unable to translate the full spectrum and expression of professional competence and, in the end, will also limit the consolidation or broadening of the spectrum of nurses’ professional practice.

Identifying and accessing relevant experts for a Delphi study can be challenging, necessitating pre-defined selection criteria [9,11,17,42]. This scoping review suggests that researchers should maintain strict control over the selection process, particularly by verifying that experts meet the eligibility criteria. Direct contact by researchers is considered the most rigorous approach [9]. This contact may be facilitated through personal knowledge, referrals from existing experts, or organisations in the field, using a controlled snowball method. Under this method, potential experts are verified for eligibility by the research team [84]. To ensure the validity of the results in Delphi studies carried out in the field of professional competence, it is crucial to avoid uncontrolled expert referrals or admissions [29].

When experts are identified through organisations, such as professional bodies, challenges arise in accessing them and verifying eligibility. In such cases, the organisation first approaches the expert. Only if the expert shows interest does the research team make direct contact [84]. It is essential to provide accurate and necessary information to the organisation to facilitate this process, clearly outlining the study’s scope and objectives while reserving detailed discussions for later direct interactions.

Attention to the control and personalisation of invitations is crucial, along with careful consideration of the expertise of Delphi panel members. Effective management in these areas correlates with greater panel cohesion and stability across rounds, reducing attrition rates. Studies included in this scoping review indicate that less researcher involvement in expert access and recruitment often leads to higher abandonment rates [59,69,75,78]. Researchers should personally contact experts and issue invitations, emphasising their importance in encouraging commitment [29,84]. For smaller panels, methods such as phone calls, face-to-face meetings (where possible), or tailored electronic invitations should be employed to maximise involvement [9]. This personalised approach is vital for success, given the high demands placed on participants in Delphi studies [9,29,84].

In the studies analysed, email was the most common method for contacting experts. Researchers used it to formally invite experts, detailing the study’s scope, purpose, objectives, and procedures [9]. However, while email is widely used, it has potential drawbacks, such as losing important communications among a high volume of messages [84].

There is no definitive guidance on the ideal number of experts for a panel [9,29]. However, the goal should include sufficient experts to capture a broad range of perspectives and understandings necessary for a comprehensive analysis of the phenomenon being studied [10,11,35,38,39,41,42,83,84]. In nursing research on professional competence, panels should include nursing educators, researchers, methodologists, psychologists, nurse managers, specialist/advanced practice nurses, and nurses from diverse clinical contexts. Additionally, involving nurses from professional regulatory bodies, nursing associations, and health policy-defining structures is beneficial. Non-nursing experts with relevant contextual knowledge may also be valuable [42,79,84]. Professional experience, including clinical practice and teaching backgrounds, should be considered [42,79]. Time criteria should be defined, such as years of teaching specific content or bibliometric indicators like published articles and conference presentations [42]. Panels should avoid including participants with conflicts of interest or those selected purely for convenience [84].

The detailed and rigorous definition of eligibility criteria for selecting experts in a Delphi panel is paramount in studying professional competence in nursing. Establishing demanding criteria demonstrates the researchers’ commitment to ensuring that the panel members are true experts on the topic and provides the credibility and quality of the information generated throughout the different iterations of the Delphi process. This meticulous approach is crucial to minimising bias and maximising the validity of the results, ensuring that the conclusions will reflect the expertise needed to address the issues under study.

Some studies have used the explicit commitment of experts to participate in all Delphi panel phases as a selection criterion in expert selection [38]. Although assessing willingness to meet this condition initially may seem beneficial, it introduces uncertainties beyond the control of both the researcher and the expert. Additionally, it could place undue pressure on experts and raise ethical concerns about participation and self-determination. Since participants, including panel experts, have the right to withdraw at any time, avoiding this criterion in expert selection is advisable.

In Delphi studies, the number of iterations often varies based on the study’s design. Studies not based on literature reviews, which lack an initial set of organised findings and use open questions or interviews to start, typically require more rounds [9,29,60,65,70,77,78]. Conversely, studies supported by literature reviews that use structured questionnaires with closed questions generally reach consensus in fewer rounds [29,61,64,68,71,74]. This distinction is crucial for researchers to consider during planning, as Delphi studies often take extended periods to complete [25,39]. The choice of data collection method, especially in the initial round, and the nature of the questions posed, along with the analysis and processing procedures, significantly affect the consensus-building pace [9,29]. It is important to note that while methodological choices primarily drive the time factor, this should not compromise study quality for expedience [29], especially when studying a field as complex as professional competence, researchers should strive to explore, to the best of their ability and in as much depth as possible, the understandings of the different experts on the topic under study, enriching, on the one hand, the panoply of elements made available to researchers, while at the same time enabling experts to confront (albeit anonymised) perspectives different from their own, which could have an impact on the course of the iterations that take place.

The instrument for the initial round should be divided into three main sections [17,79,84]. The first section should define the study’s scope, objectives, the expert’s role, and the importance of their participation. It should also include instructions for completing the questionnaire and informed consent form [9,17,38,79,84]. The second section should gather essential data to characterise the panel of experts, focusing on necessary sociodemographic details to assess the group’s expertise and knowledge level [11,17,29,36,38,83]. The third section contains the questions, whether open-ended or structured, aimed at exploring the subject or seeking consensus. In subsequent rounds, the instrument should consist of two sections: one with specific instructions for that round, especially if the initial round involved open-ended questions, and the other with controlled feedback and the questionnaire items or statements [29].

The literature review highlights considerable variation in the setup of initial Delphi study rounds, ranging from closed-question questionnaires with Likert-type scales [38,79,83] to open-ended inquiries and interviews with panel experts [9,29,38]. While exploratory approaches are necessary in cases of limited, contradictory, or inconsistent evidence, researchers should, where possible, support these methods with existing evidence to enhance result validity [9,79]. Despite constrained evidence, efforts should focus on using it to frame open-ended questions or exploratory interviews rather than relying solely on expert opinions [9,17,36].

Researchers can always include open-ended questions regardless of how direct the questions are, including those using Likert-type scales. These allow experts to share opinions, offer suggestions, or justify specific viewpoints, helping to address uncertainties [9,10,17,29,38,79]. The information gathered and controlled feedback—the only form of communication among panel members—can reveal diverse perspectives, this being fundamental in a process designed to define a core body of professional competencies or develop an instrument for assessing and quantifying professional competence. This approach fosters deeper discussion, builds consensus, and enhances the study’s validity [9,10,29,38].

It is crucial to acknowledge that expert participation can be demanding, as it involves navigating various steps, including analysing instructions and controlled feedback from previous phases, which are vital to achieving the study’s objectives [9,10,17,29,38,84]. A 14-day timeframe for completing the instrument is generally reasonable in the reviewed studies. Shorter periods may place undue pressure on participants, while overly long deadlines could reduce the urgency of responding, potentially leading to lower response rates [9].

Attrition in Delphi studies is a significant risk to the quality and validity of outcomes [29]. Analysis shows that the impact of losing panel experts becomes more critical as the initial panel size decreases. Larger panels can absorb a higher dropout rate [78], but when the panel is small [59,69], increased attrition can disrupt the study’s dynamics and weaken the robustness of its findings. This is because attrition undermines the panel’s representativeness and the depth of understanding required for consensus [29,42].

Researchers must actively engage experts to reduce attrition and sample loss [9,29,42]. Acknowledging their crucial role from the invitation stage helps experts feel valued rather than merely transient contributors [42]. This appreciation should continue throughout the study [9,29,42]. Researchers should show understanding regarding response delays, be flexible with deadlines, and consider the professionals’ multiple responsibilities [9,42]. It should be borne in mind that the potential mortality of the sample in Delphi studies carried out in the field of professional competence could harm the validity of the results insofar as a panel initially set up to bring together all the perspectives and opinions on a subject, when it loses experts due to withdrawal, ceases to be truly representative, with all that this represents in terms of not expressing the proper level of expertise required.

A critical methodological challenge in the Delphi method is defining consensus. Researchers must specify how the agreement will be measured and what threshold will indicate consensus [38,48], given the lack of clear methodological guidelines [29,45,83]. The absence of a universal consensus definition can be advantageous, allowing researchers to set criteria tailored to their study [9,33]. However, it is essential to establish and clearly communicate the consensus criteria before the Delphi panel begins and to maintain this consistency throughout the study to ensure transparency [9,11,36]. Many studies reviewed lacked clarity on this criterion, which could raise concerns about possible adjustments made for convenience. This step is crucial to avoid undermining the study’s credibility.

The studies in this review used various approaches to define consensus, primarily focusing on the percentage of agreement required [9]. While respecting the discretion of research teams, aiming for a consensus threshold of at least 75% is advisable [9], as higher percentages indicate more substantial consensus [79]. Additionally, researchers should evaluate the stability of responses across rounds using measures such as the coefficient of variation or the non-parametric X^2^ test [10,17,25,48,83,85].

Researchers using the Delphi technique must thoroughly document the ethical and legal procedures they follow [41,79], including how data from experts will be processed, stored, and disposed of. This extends beyond informed consent forms and confidentiality safeguards [29,30]. Additional issues arise with the shift to digital environments, such as using email for questionnaires and digital platforms for data collection. These should be detailed in the research protocol and reports, including ensuring that data collection platforms comply with data protection regulations and address participant location traceability. Researchers should also specify how information will be processed, stored, and destroyed, including timing and methods [41,81,86,87].

A key criticism of the Delphi method highlighted in several studies is the insufficient detail in reporting, which raises concerns about methodological rigour, reliability, and validity. Researchers should prioritise detailed reporting and transparency, particularly in justifying methodological choices. This includes clearly describing how experts are accessed and selected, eligibility criteria, the number of invitations issued, and the instruments and methods used to gather expert opinions. Additionally, researchers should detail the organisation and duration of rounds, response times, the operationalisation of controlled feedback (both qualitative and quantitative), consensus criteria, and the study’s conclusion criteria. The information should enable readers to replicate the study precisely. Researchers are encouraged to use the ACCORD guideline (ACcurate COnsensus Reporting Document) to address these issues and standardise reporting. This global tool promotes rigorous and transparent reporting of consensus methods in health research. By following the ACCORD guideline, researchers can provide comprehensive insights into consensus methods, potentially improving patient outcomes. These guidelines were developed based on best practices from the EQUATOR network, including a systematic review and consensus exercise [88].

### Limitations

This review has a few limitations. Firstly, some relevant publications might be missing due to the inability to obtain necessary clarifications from the authors. The information in these publications required additional context that could not be provided, leading to their exclusion.

Secondly, the review did not assess the methodological quality of the included studies, which, while optional, could be seen as a constraint. This choice focused on exploring and mapping the methodological approaches used in Delphi studies. While not evaluating methodological quality introduces some fragility, it is believed that this approach allowed for a more comprehensive inclusion of various methodological choices, enriching the results, discussion, and analysis.

Despite these limitations, the review’s exploratory and critical nature has enabled it to capture a broad range of relevant evidence. It offers a thorough overview of the Delphi technique’s use in nursing competence and presents various methodological options for researchers considering this approach.

## 5. Conclusions

To the best of our knowledge, this scoping review is the first to focus specifically on the use of the Delphi method in the study of professional competence in nursing. It emphasises its application in defining core competency frameworks and developing or adapting instruments for assessing nursing competence. Our findings highlight the suitability and usefulness of the Delphi technique in this field of study. Despite longstanding limitations and criticisms associated with the method, there are crucial methodological procedures that researchers must safeguard and justify, both during the project and in reporting the results, to ensure and demonstrate the study’s rigour.

To further address the limitations and critiques of the Delphi technique, it is essential to uphold its fundamental principles, including participant anonymity (or “quasi-anonymity”), controlled feedback, and statistical analysis of the group’s responses. Additionally, careful consideration must be given to the process of accessing, selecting, and forming the panel of experts, as this is vital to the integrity of such studies. It is recommended that researchers aim to assemble a panel that captures the diversity of opinions on the topic under investigation. This ensures that the consensus reached reflects a broad spectrum of viewpoints rather than a narrow segment. Furthermore, the level of expertise and specialisation of the experts should be carefully considered, with objective criteria defined for their selection and recruitment. Similarly, clarity is required in the structuring and execution of the rounds, including a predetermined and consistently applied definition of the consensus criteria, which should remain stable throughout the study.

Finally, to address criticisms regarding the lack of rigour and detail in the reporting of Delphi studies, it is crucial to standardise and thoroughly document the methodological choices made. In this regard, the adoption of the recently published ACCORD guideline (Accurate Consensus Reporting Document) is strongly recommended.

By attending to these aspects, researchers can enhance their studies’ reliability, validity, and methodological robustness, thereby reinforcing the Delphi technique and mitigating its perceived shortcomings.

## Figures and Tables

**Table 1:** Search strategy used in MEDLINE (via EBSCO).

Search No.	Search Terms and Expressions	Results
S1	MM “Delphi Technique” OR TI “delphi” OR AB “delphi” OR TI “delphi technique” OR AB “delphi technique” OR TI “delphi survey” OR AB “delphi survey” OR TI “delphi consensus” OR AB “delphi consensus” OR TI “delphi study” OR AB “delphi study” TI “delphi method” OR AB “delphi method” OR TI “expert consensus method” OR AB “expert consensus method” OR TI “modified nominal group technique” OR AB “modified nominal group technique” OR TI “forecasting method” OR AB “forecasting method” OR TI “decision-making method” OR AB “decision-making”	185,322
S2	TI “assessment scale” OR AB “assessment scale” OR TI “evaluation scale” OR AB “evaluation scale” OR TI “assessment instrument development” OR AB “assessment instrument development” OR TI “evaluation tool” OR AB “evaluation tool” OR TI “scale development” OR AB “scale development” OR TI “factor analysis” OR AB “factor analysis” OR TI “instrument design” OR AB “instrument design” OR TI “instrument development” OR AB “instrument development” OR TI “instrument validation” OR AB “instrument validation” OR TI “item analysis” OR AB “item analysis” OR TI “psychometric instrument development” OR AB “psychometric instrument development” OR TI “psychometric testing” OR AB “psychometric testing” OR TI “questionnaire development” OR AB “questionnaire development” OR TI “reliability testing” OR AB “reliability testing” OR TI “survey development” OR AB “survey development” OR TI “validation studies” OR AB “validation studies”	85,186
S3	MM “Professional Competence” OR TI “professional competence” OR AB “professional competence” OR TI “competenc*” OR AB “competenc*” OR TI “knowledge” OR AB “knowledge” OR TI “proficiency” OR AB “proficiency” OR TI “expertise” OR AB “expertise” OR TI “capability” OR AB “capability” OR TI “ability” OR AB “ability” OR TI “skill*” OR AB “skill*”	2,309,012
S4	MM “Nursing” OR TI “nurs*” OR AB “nurs*” OR TI “nursing practice” OR AB “nursing practice” OR TI “nursing research” OR AB “nursing research” OR TI “nursing education” OR AB “nursing education” OR TI “nursing management” OR AB “nursing management” OR TI “nursing care” OR AB “nursing care” OR TI “nursing interventions” OR AB “nursing interventions”	526,118
S5	S1 AND S2 AND S3 AND S4	136

**Table 2:** Steps, processes and methodological options used in the different Delphi studies included in the literature review.

Steps and Procedures	Methodological Options	Study
Preparatory procedures	- Literature review	[60,61,62,63,64,67,68,72,73,74]
	- Literature review and expert consultation (focus groups and discussion groups)	[59,60,61,63,66,71,73]
	- Deliberation among the research team regarding the components to be presented for evaluation to the Delphi panel	[66,67,71,75]
	- No preparatory procedures	[65,70,76,77]
Expert access procedures	- The experts were recruited conveniently through direct and exclusive contact initiated by research team members	[60,63,77]
	- Recruitment by referencing and snowball method	[59,65,67]
	- Experts recruited by identifying them through online platforms of organisations pertinent to the study area of specific competence	[59,75]
	- Recruitment through direct contact with relevant organisations in certain areas of expertise	[63,71,73]
	- Recruitment through electronic newsletters, institutional online websites, and scientific meetings	[69,75]
	- Recruitment by reviewing papers relevant to a particular area of expertise	[77]
Call for expert participation procedures	- Email	[59,65,69,70,71,73,75]
	- Telephone contact and face-to-face contact promoted by the researchers	[69]
	- Face-to-face contact by reference elements identified by the research team in the professional practice contexts from which the experts came	[72]
Expert selection procedures	- Selection process involved representing various facets, sensitivities, and current understandings of the topic	[69,72,76]
	- Selection was conducted based on criteria related to the specificity and nature of the position held by the expert	[59,60,61,64,65,66,67,69,70,71,73,74,76,78,79]
	- Selection process considered geographical and cultural differences as criteria	[59,67,77]
	- Selection based on the experts’ professional experience relevant to the topic under study	[60,62,63,64,66,67,68,69,71,72,73,77]
	- Selection based on the expert’s express indication of willingness to participate in the study	[60,67,70]
Instrumentation	- Electronic questionnaire	[59,61,69,70,72,73,74,76]
	- Postal questionnaire	[77,78]
	- Semi-structured interview (telephone, face-to-face or group dynamics) and questionnaire	[60,63,65,66]
Data analysis	- Content analysis, establishing a structure of categories and thematic areas (domains and subdomains) or statements	[65,69,72,77]
	- Absolute frequencies and relative frequencies, mean and standard deviation	[60,65,70,73,78]
	- Content Validity Index (CVI)	[61,62,63,66,67,68,69,70,72,73,74,76]
	- Mean and standard deviation	[63,70,73,75,78]
	- Expert coordination coefficient (Kendall’s W) and coefficient of variation (CV)	[60,64,66,67,71]
	- Weighted Kappa coefficient (Kw)	[67]

## Supplementary Materials

The following supporting information can be downloaded at: https://www.mdpi.com/article/10.3390/healthcare12171757/s1, Table S1: Preferred Reporting Items for Systematic Reviews and Meta-Analyses for Scoping Reviews (PRISMA-ScR) Checklist; Table S2: Abstract reporting checklist required from the Preferred Reporting Items for Systematic Reviews and Meta-Analyses for Scoping Reviews (PRISMA-ScR); Table S3: Search strategy used in the PubMed, Web of Science, CINAHL (via EBSCO) and MEDLINE (via EBSCO) databases, on 30 April 2023; Table S4: Characteristics main findings extracted from the primary studies included in the scoping review.
