# Supplementary material for: Delphi Technique on Nursing Competence Studies: A Scoping Review
Source: Healthcare (Basel). 2024 Sep 3;12(17):1757. doi: 10.3390/healthcare12171757 (PMC11395531; doi:10.3390/healthcare12171757)
Supplement: Supplementary file 1 [file healthcare-12-01757-s001.zip › Table_S3.pdf]

Table S3. Search strategy used in the PubMed, Web of Science, CINAHL (via EBSCO) and MEDLINE (via EBSCO) databases, on April 30, 2023.

SEARCH NO.	SEARCH TERMS AND EXPRESSIONS	RESULTS
	**PubMed**	
#1	"Delphi Technique"[Mesh] OR "delphi"[tiab] OR "delphi technique*"[tiab] OR "delphi survey"[tiab] OR "delphi consensus"[tiab] OR "delphi study"[tiab] OR "delphi method*"[tiab] OR "expert consensus method"[tiab] OR "modified nominal group technique"[tiab] OR "forecasting method"[tiab] OR "decision-making method"[tiab]	16,634
#2	"assessment scale"[tiab] OR "evaluation scale"[tiab] OR "assessment instrument development"[tiab] OR "evaluation tool"[tiab] OR "scale development"[tiab] OR "factor analysis"[tiab] OR "instrument design"[tiab] OR "instrument development"[tiab] OR "instrument validation"[tiab] OR "item analysis"[tiab] OR "psychometric instrument development"[tiab] OR "psychometric testing"[tiab] OR "questionnaire development"[tiab] OR "reliability testing"[tiab] OR "survey development"[tiab] OR "validation studies"[tiab]	88,934
#3	"Professional Competence"[Mesh] OR "professional competence"[tiab] OR competenc*[tiab] OR knowledge[tiab] OR proficiency[tiab] OR expertise[tiab] OR capability[tiab] OR ability[tiab] OR skill*[tiab]	2,378,408
#4	"Nursing"[Mesh] OR "nurs*"[tiab] OR "nursing practice"[tiab] OR "nursing research"[tiab] OR "nursing education"[tiab] OR "nursing management"[tiab] OR "nursing care"[tiab] OR "nursing interventions"[tiab]	651,680
#5	#1 AND #2 AND #3 AND #4	78
	**Web of Science**	
#1	(((((((((TS=("delphi")) OR TS=("delphi technique")) OR TS=("delphi survey")) OR TS=("delphi consensus")) OR TS=("delphi study")) OR TS=("delphi method")) OR TS=("expert consensus method")) OR TS=("modified nominal group technique")) OR TS=("forecasting method")) OR TS=("decision-making method")	37,476
#2	(((((((((((((((((TS=("assessment scale")) OR TS=("evaluation scale")) OR TS=("assessment instrument development")) OR TS=("evaluation tool")) OR TS=("scale development")) OR TS=("factor analysis")) OR TS=("instrument design")) OR TS=("instrument development")) OR TS=("instrument validation")) OR TS=("item analysis")) OR TS=("psychometric instrument development")) OR TS=("item analysis")) OR TS=("psychometric instrument development")) OR TS=("psychometric testing")) OR TS=("questionnaire development")) OR TS=("reliability testing")) OR TS=("survey development")) OR TS=("validation studies")	164,533
#3	(((((((TS=("professional competence")) OR TS=("competenc*")) OR TS=("knowledge")) OR TS=("proficiency")) OR TS=("expertise")) OR TS=("capability")) OR TS=("ability")) OR TS=("skill*")	4,555,781
#4	((((((TS=("nurs*")) OR TS=("nursing practice")) OR TS=("nursing research")) OR TS=("nursing education")) OR TS=("nursing management")) OR TS=("nursing care")) OR TS=("nursing interventions")	415,287
#5	#1 AND #2 AND #3 AND #4	86
	**CINAHL**	
S1	MM "Delphi Technique" OR TI "delphi" OR AB "delphi" OR TI "delphi technique" OR AB "delphi technique" OR TI "delphi survey" OR AB "delphi survey" OR TI "delphi consensus" OR AB "delphi consensus" OR TI "delphi study" OR AB "delphi study" TI "delphi method" OR AB "delphi method" OR TI "expert consensus method" OR AB "expert	77,364

	consensus method" OR TI "modified nominal group technique" OR AB "modified nominal group technique" OR TI "forecasting method" OR AB "forecasting method" OR TI "decision-making method" OR AB "decision-making"	
S2	TI "assessment scale" OR AB "assessment scale" OR TI "evaluation scale" OR AB "evaluation scale" OR TI "assessment instrument development" OR AB "assessment instrument development" OR TI "evaluation tool" OR AB "evaluation tool" OR TI "scale development" OR AB "scale development" OR TI "factor analysis" OR AB "factor analysis" OR TI "instrument design" OR AB "instrument design" OR TI "instrument development" OR AB "instrument development" OR TI "instrument validation" OR AB "instrument validation" OR TI "item analysis" OR AB "item analysis" OR TI "psychometric instrument development" OR AB "psychometric instrument development" OR TI "psychometric testing" OR AB "psychometric testing" OR TI "questionnaire development" OR AB "questionnaire development" OR TI "reliability testing" OR AB "reliability testing" OR TI "survey development" OR AB "survey development" OR TI "validation studies" OR AB "validation studies"	37,153
S3	MM "Professional Competence" OR TI "professional competence" OR AB "professional competence" OR TI "competenc*" OR AB "competenc*" OR TI "knowledge" OR AB "knowledge" OR TI "proficiency" OR AB "proficiency" OR TI "expertise" OR AB "expertise" OR TI "capability" OR AB "capability" OR TI "ability" OR AB "ability" OR TI "skill*" OR AB "skill*"	580,234
S4	MM "Nurses" OR TI "nurs*" OR AB "nurs*" OR TI "nursing practice" OR AB "nursing practice" OR TI "nursing research" OR AB "nursing research" OR TI "nursing education" OR AB "nursing education" OR TI "nursing management" OR AB "nursing management" OR TI "nursing care" OR AB "nursing care" OR TI "nursing interventions" OR AB "nursing interventions"	626,027
S5	S1 AND S2 AND S3 AND S4	311
	**MEDLINE**	
S1	MM "Delphi Technique" OR TI "delphi" OR AB "delphi" OR TI "delphi technique" OR AB "delphi technique" OR TI "delphi survey" OR AB "delphi survey" OR TI "delphi consensus" OR AB "delphi consensus" OR TI "delphi study" OR AB "delphi study" TI "delphi method" OR AB "delphi method" OR TI "expert consensus method" OR AB "expert consensus method" OR TI "modified nominal group technique" OR AB "modified nominal group technique" OR TI "forecasting method" OR AB "forecasting method" OR TI "decision-making method" OR AB "decision-making"	185,322
S2	TI "assessment scale" OR AB "assessment scale" OR TI "evaluation scale" OR AB "evaluation scale" OR TI "assessment instrument development" OR AB "assessment instrument development" OR TI "evaluation tool" OR AB "evaluation tool" OR TI "scale development" OR AB "scale development" OR TI "factor analysis" OR AB "factor analysis" OR TI "instrument design" OR AB "instrument design" OR TI "instrument development" OR AB "instrument development" OR TI "instrument validation" OR AB "instrument validation" OR TI "item analysis" OR AB "item analysis" OR TI "psychometric instrument development" OR AB "psychometric instrument development" OR TI "psychometric testing" OR AB "psychometric testing" OR TI "questionnaire development" OR AB "questionnaire development" OR TI "reliability testing" OR AB "reliability testing" OR TI "survey development" OR AB "survey development" OR TI "validation studies" OR AB "validation studies"	85,186

S3	MM "Professional Competence" OR TI “professional competence” OR AB “professional competence” OR TI “competenc*” OR AB “competenc*” OR TI “knowledge” OR AB “knowledge” OR TI “proficiency” OR AB “proficiency” OR TI “expertise” OR AB “expertise” OR TI “capability” OR AB “capability” OR TI “ability” OR AB “ability” OR TI “skill*” OR AB “skill*”	2,309,012
S4	MM "Nursing" OR TI “nurs*” OR AB “nurs*” OR TI “nursing practice” OR AB “nursing practice” OR TI “nursing research” OR AB “nursing research” OR TI “nursing education” OR AB “nursing education” OR TI “nursing management” OR AB “nursing management” OR TI “nursing care” OR AB “nursing care” OR TI “nursing interventions” OR AB “nursing interventions”	526,118
S5	S1 AND S2 AND S3 AND S4	136
**TOTAL**		**611**
