# Supplementary material for: Delphi Technique on Nursing Competence Studies: A Scoping Review
Source: Healthcare (Basel). 2024 Sep 3;12(17):1757. doi: 10.3390/healthcare12171757 (PMC11395531; doi:10.3390/healthcare12171757)
Supplement: Supplementary file 1 [file healthcare-12-01757-s001.zip › Table_S4.pdf]

Table S4. Characteristics main findings extracted from the primary studies included in the scoping review.

Author(s) and year	Source	Title	Country	Study's aim	INE	FNE	EDOR	TNR	Main findings
Irvine (2005) [78]	Journal of Clinical Nursing	Exploring district nursing competencies in health promotion: the use of the Delphi technique	United Kingdom	To explore the competence profile of district nurses in health promotion.	181	56	-69,1%	3	¬ The first stage of the study involved assembling the panel of experts, which was accomplished using the snowball method for expert recruitment. The panel was intentionally diverse, focusing on regional representativeness, comprising doctors, nurses (across various levels and specialities), managers, university lecturers, nursing educators, and nursing students.<br>¬ In the initial round, items were generated through two open-ended questions. Subsequent rounds utilised closed questions with Likert-type scales (1-5). All instruments were distributed and collected via postal mail.<br>¬ Mean scores were employed to gauge the panel group's opinions, while standard deviations (SDs) were utilised to indicate the level of agreement within the panel. A standard deviation 1.2 was determined as the appropriate threshold for establishing consensus.
Jirwe, Gerrish, Keeney and Emami (2009) [77	Journal of Clinical Nursing	Identifying the core components of cultural competence: findings from a Delphi study	Sweden	To identify the essential components of cultural competence from the Swedish perspective.	24	20	-16,7%	4	¬ The Delphi study was conducted based on insights gathered through interviews with a panel of experts. This panel comprised a diverse group within the professional nursing community, including nurses from various levels and specialities, higher education professors, nurse educators, and researchers with knowledge and experience in nursing within multicultural contexts. Clear criteria were established to assess expertise and familiarity with multicultural issues, and experts were identified through scientific reviews and the snowball method. Objective selection and recruitment criteria were defined for each type of expert.<br>¬ In the initial round, semi-structured individual interviews ranged from 20 to 45 minutes. Participants were prompted to identify specific knowledge, skills, and attitudes necessary for nurses when caring for patients from diverse cultural backgrounds and were encouraged to elaborate on their responses. Content analysis of these interviews generated statements for subsequent rounds. Subsequent rounds utilised questionnaires with closed questions, employing Likert-type scales. Experts were consistently provided with opportunities to offer suggestions and address any uncertainties.<br>¬ A consensus level of 75 per cent was established as the threshold for agreement.
Chang, Gardner, Duffiel and Ramis (2010) [76]	Journal of Advanced Nursing	A Delphi study to validate an advanced practice nursing tool	Australia	To validate an instrument aimed at delineating the boundaries of the role of advanced practice	16	15	-6,3%	3	¬ The panel of experts consisted exclusively of nurses from diverse practice areas, including clinicians, educators, managers, nurse specialists, and directors, representing rural, remote, and metropolitan environments. Panel members were required to possess comprehensive knowledge and familiarity with the parameters of professional nursing practice and the

				nurses within an international health services framework, utilising the Delphi technique.					workforce requirements of the healthcare service, as well as be credible in their respective areas of activity.<br>¬ Round 1 commenced with closed questions employing Likert-type scales (1-5), with an opportunity for panellists to provide comments. Responses and comments from the first round were compiled and distributed to each panellist, along with additional information following their feedback.<br>¬ Considering the panellists ' representation from diverse regions, a combined email and online data collection method was chosen.<br>¬ At the study's outset, it was established that activities rated with 3 or more points and a content validity index (CVI) of 0.75 or higher would be deemed an acceptable level of consensus. The CVI was calculated based on the percentage of panellists who rated items as four or more points on the Likert scale (agree or strongly agree) for each domain or activity listed on the scale.
Hoyt, Coyne, Ramirez, Peard, Gisness and Gacki-Smith (2010) [75]	Journal of Emergency Nursing	Nurse Practitioner Delphi Study: competencies for practice in emergency care	United States of America	Identify the essential competencies of the nurse practitioner for practice in emergency care	128	52	-59,4%	3	¬ An extensive literature review was conducted, resulting in the development of an initial list comprising 107 competencies.<br>¬ Participants were recruited through various channels, including the website, newsletters, and scientific conferences, utilising flyers, advertisements, and an advanced practice networking session. The primary criterion for admitting experts was their current or recent professional experience in advanced practice within an urgent or emergency context.<br>¬ All rounds of the study utilised closed questions with Likert-type scales (1-6). In the first round, participants were asked to provide suggestions for rewording competency statements that they found unclear and to identify any competencies they believed were omitted from the questionnaire. Additionally, demographic questions were included in the first-round questionnaire.<br>¬ A competency statement was included in the final list if 80 per cent or more of the Delphi participants rated the competency as highly important, denoted by a minimum rating of 4 on a 6-point scale.
Van Hecke, Goeman, Beeckman, Heinen and Defloor (2011) [74]	Journal of Advanced Nursing	Development and psychometric evaluation of an instrument to assess venous leg ulcer lifestyle knowledge among nurses	Belgium	To develop and test the psychometric properties of an instrument to assess nurses' knowledge of lifestyle in the case of venous leg ulcers.	9	9	0,0%	2	¬ Determining topics to be included in the tool was grounded in an exhaustive literature review. Multiple-choice questions and clinical cases for the instrument were developed subsequently. Multiple-choice questions were designed to evaluate factual knowledge, while clinical cases aimed to assess more complex cognitive skills.<br>¬ Experts with expertise in leg ulcer treatment or leg ulcer research were enlisted for evaluation. They assessed the questions and answers regarding accuracy, clarity, readability, and relevance.<br>¬ The Content Validity Index (CVI) was employed to gauge the experts' consensus regarding the accuracy of the questions.

Lakanmaa, Suominen, Perttilä, Puukka and Leino-Kilpi (2012) [72]	Intensive and Critical Care Nursing	Competence requirements in intensive and critical care nursing – Still need definition? A Delphi study	Finland	To identify the criteria for nurses' competence in intensive care and develop a tool for assessing nurses' competence in intensive care.	45	45	0,0%	2	¬ Before convening the Delphi panel, a comprehensive literature review was conducted.<br>¬ The composition of the expert panel aimed for a broad representation of nurses and doctors, including individuals with varying degrees of professional experience. Objective selection criteria were established based on job function, position, total professional experience, and experience in their current role, with specific criteria tailored to each professional group. Each clinical practice setting had a designated contact person who collaborated with the research team to recruit experts, who were contacted via postal mail.<br>¬ In the first round, data was collected on expert characteristics such as age, gender, educational qualifications, professional experience in intensive care, other healthcare sector experience, and additional training in intensive care/critical care nursing. Experts were also asked to provide their opinions on the question: "In your opinion, what defines the competence of intensive care nurses?"<br>¬ Subsequent rounds involved experts ranking the domains and subdomains of competence identified in the first round using a Likert-type scale (1-5). A predetermined level of consensus was established beforehand.<br>¬ To assume consensus, two conditions needed to be met: 1) an average score of at least 4 and 2) a consensus percentage of at least 80 per cent. Consensus percentage was calculated by categorising values 1-3 as "not important" (0) and values 4-5 as "important" (1).
Liu, Curtis and Crookes (2014) [73]	Journal of Hospital Infection	Identifying essential infection control competencies for newly graduated nurses: a three-phase study in Australia and Taiwan	Australia and Taiwan	To identify the essential infection prevention and control competencies of newly qualified nurses.	122	80	-34,4%	4	¬ For phase I (developing a questionnaire for the first round of the study), three steps were followed: 1) review of relevant literature to identify a preliminary list of possible infection control competencies for newly qualified nurses; 2) development of the first round questionnaire based on the review of relevant references; and 3) validation of the first round questionnaire to ensure that the instrument was well developed. A reference group of experts (N = 4; two from Australia and two from Taiwan) collaborated to ensure that the first questionnaire round applied to both countries' healthcare contexts.<br>¬ A panel of experts was selected for this study, targeting members of infection control organisations in Australia and Taiwan responsible for disseminating current infection prevention and control practices. Academics from nursing schools in Australia and Taiwan were also selected.<br>¬ To represent expertise from across the country, relevant infection control organisations and nursing schools in Australia and Taiwan were first identified by the researcher. Selective sampling was used to identify nominated health professionals and nursing lecturers who met at least one

									of the following criteria: 1) have published articles on infection control in medical, healthcare and nursing journals; 2) have presented posters or papers at infection control conferences; 3) have delivered lectures on infection control; or 4) have extensive knowledge and experience and are considered by their peers to be experts in infection control.<br>¬ Each round of the survey was carried out simultaneously in both countries. In the first round, the experts were asked to present as many relevant questions as possible, giving their opinions on the subject of study.<br>¬ Once consensus had been reached on the items in the original round I questionnaire, subsequent rounds used a modified questionnaire from which the items that had already reached consensus were removed. During the Delphi rounds, the researcher presented the panel of experts with relevant information on infection control and asked the panel to make judgements and comments on the items presented until a consensus was reached. The initial questionnaire for the first round included open-ended questions to collect qualitative data and questions with a 5-point Likert scale to collect quantitative data. The following rounds included closed questions and spaces for comments.<br>¬ Consensus for each item of the infection control competencies was established as >80% of the panellists rated it 4 or 5 regarding applicability and importance.
Bing-Jonsson, Bjork, Hofoss, Kirkevold and Foss (2015) [65]	International Journal of Older People Nursing	Competence in advanced older people nursing: development of 'Nursing older people - Competence evaluation tool'	Norway	Carry out a Norwegian-based Delphi study to develop the substantive content of a new instrument for measuring the competencies of nursing staff most relevant to meeting the current needs of elderly patients.	42	39	-7,1%	3	¬ The experts were carefully selected to encompass a wide range of perspectives and expertise in the field of eldercare, including clinicians specialising in eldercare, leaders or administrators in community care services, nursing professors focusing on eldercare, nursing researchers, and individuals from relevant user organisations. They were regarded as "informed individuals" and "experts in their field" with substantial knowledge in eldercare.<br>¬ Utilising the snowball technique, experts were individually selected, with all participants recommended by other experts to ensure credibility and expertise. The diversity of participants was deemed crucial to ensure the validity of the results, encompassing various professions, locations, ages, and genders to capture a comprehensive range of perspectives on nurses caring for older people.<br>¬ Initial contact with participants was made via email, outlining the purpose and requirements of participating in the study.<br>¬ The first round involved conducting 42 individual, semi-structured telephone interviews, each lasting approximately 30 minutes. The saturation of categories and items was achieved after approximately ten interviews, identifying 110 items across 14 categories through categorisation and

									coding of meaning. In the second round, experts responded to an electronic questionnaire via email, comprising items developed from the qualitative analysis in Round 1. Respondents rated the items based on their relevance to nurses caring for older people using a 5-point Likert-type scale. They were also encouraged to provide additional comments whenever necessary.<br>¬ A consensus level of 90 per cent was established as the cut-off point, with scores of 4 (very relevant) and 5 (decisive) indicating the panel's agreement with a given item.
Fan, Gui, Xi and Qiao (2016) [66]	International Journal of Nursing Sciences	Core competence evaluation standards for emergency nurse specialist: Developing and testing psychometric properties	China	Developing a tool to assess the essential competencies of nurses specialising in urgent/emergency care.	17	17	0,0%	2	¬ A discussion group was convened to develop the framework of core competencies. Subsequently, an expert survey form was prepared to conduct interviews with consultants. The data collected from these interviews was subjected to statistical analysis. Through the expert interviews, relevant concepts and competence requirements were determined.<br>¬ To establish the set of indices for the assessment standards, a comprehensive literature review was conducted, encompassing authoritative documents from various international bodies such as the International Council of Nursing, as well as professional registration requirements for nurses in China, the USA, the UK, Australia, Hong Kong, and Taiwan. The research group defined the key concepts and the first and second-level dimensions in the draft evaluation index system.<br>¬ The project included eight first-level dimensions, 35 second-level dimensions, and 180 items.<br>¬ Seventeen experts, comprising 15 expert nurses and two doctors from hospitals in Shanghai, were selected for participation. These experts were required to possess at least a bachelor's degree, hold a high academic reputation in nursing, and have more than ten years of professional experience.<br>¬ The Delphi method was conducted via electronic mail. Experts utilised a 5-point Likert rating scale for evaluation, ranging from 1 (very unimportant) to 5 (very important). The selection of index dimensions and items was based on the results of coefficients of variation and the average ratings. Coefficients of variation below 0.25 and means exceeding 3.5 were considered acceptable for inclusion.
Zheng, Shi, Jiang, Li and Zhang (2017) [67]	Biomedical Research	Evaluation of core competencies of nurses by novel holistic assessment system	China	Develop an integrated measurement system for assessing nurses' competencies.	34	28	-17,6%	3	¬ The study commenced with an extensive literature review and expert consultations using questionnaires and semi-structured interviews. Following a thorough analysis of the collected data and subsequent discussions and modifications, competency domains were identified to delineate the essential competencies expected of nurses accurately.

									Subsequently, the initial draft of these competency standards was formulated.<br>¬ Experts participating in the Delphi study were drawn from diverse nursing contexts within Grade III Class A hospitals—the highest level of hospitals in China—spanning various regions across the country. The selection of experts adhered to the following principles: 1) inclusion of representative and authoritative figures; 2) representation from different geographical regions of China; 3) inclusion of professionals from various levels of nursing; 4) representation from both teaching and non-teaching hospitals; and 5) inclusion of professionals from both general and specialised hospitals. Inclusion criteria comprised: 1) involvement in clinical practice, administration, teaching, or research in nursing; 2) possession of an associate's degree or higher; 3) demonstration of initiative and willingness to participate in the Delphi procedure; 4) commitment to respond to all three Delphi rounds; and 5) meeting the requisite years of professional nursing practice: 10 years or more for individuals with an associate's degree, five years or more for those with a bachelor's degree, three years or more for those with a master's degree, and two years or more for those with a doctoral degree. Additionally, three criteria were established to exclude experts deemed unsuitable for the study: 1) occupation in non-critical care nursing; 2) possession of academic qualifications lower than an associate's degree; and 3) lack of willingness to participate in the research.<br>¬ Questionnaires were distributed to the experts via email in each round, and those providing valid responses proceeded to participate as expert contributors in subsequent rounds.<br>¬ Weight and composite index methodologies were employed to gauge the importance of each competency domain. Kendall's coefficient of agreement (W) was utilised to evaluate consensus among the experts. At the same time, the content validity of the competency assessment scale was assessed based on the content validity index and inter-rater agreement.<br>¬ Considering practical implications, the project team established a minimum 80% approval rate to ensure significant agreement among Delphi experts. Kendall's coefficient of agreement (W) was employed to signify overall consensus among all experts regarding the evaluated data. A smaller P-value in the hypothesis test indicates greater consistency and concordance among expert opinions, yielding more reliable results.
Chen, Pu, Chen, Xu, Bai & Hu (2019) [68]	Clinical Nurse Specialist	Instrument development for evaluation of gerontological nurse	China	To determine the essential competencies of Chinese nurses specialising in	30	28	-6,7%	2	¬ The initial phase involved constructing a framework of essential competencies through theoretical analysis. This framework was developed based on the definition of competence within the nursing context,

		specialists core competencies in China		gerontology and to test the psychometric properties of an instrument for self-assessment of essential competencies.					encompassing knowledge, skills, personal attributes, and attitudes necessary to fulfil nursing duties following employment standards.<br>¬ The first iteration of the core competencies was distributed to 30 experts who met the inclusion criteria of possessing more than ten years of experience in geriatric nursing and holding the professional title of head nurse or higher.<br>¬ Experts evaluated the importance of each first-level domain and each second-level dimension using a 5-point Likert rating scale. Additionally, sixty-nine third-level items were assessed on a 5-point Likert scale in terms of both importance and feasibility. Furthermore, a column titled "delete, modify, and add items" was provided for experts to comment.<br>¬ An item was removed from consideration if its average importance or feasibility score equalled or fell below 3.5, if the coefficient of variation was equal to or exceeded 0.25, or if the consistency rate (percentage of choices 4 or 5) was equal to or lower than 75%.
Holanda, Marra and Cunha (2019) [69]	Revista Brasileira de Enfermagem	Professional competence of nurses in emergency services: evidence of content validity	Brazil	To verify the content validity of the identifying questions based on the theoretical-logical model of the Matrix and the Professional Competence Profile for emergency nurses.	25	18	-28,0%	4	¬ The rationale behind the expert selection process and the composition of the final panel emphasised the importance of heterogeneity among the experts. Several eligibility criteria were established: 1) completion of postgraduate training; 2) experience in emergency situations, professional competence, or both; 3) professional experience in a clinical practice setting relevant to the study's topic; 4) involvement in professional training, management, teaching, or research.<br>¬ Experts were identified through an active search on institutional websites of universities, professional nursing associations, and public organisations relevant to the topic, where individuals with the specified characteristics were located.<br>¬ Upon identification, experts were contacted via email, telephone, or in-person meetings to introduce the research project, objectives, data collection methods and procedures, study timeline, rationale for their selection as panel members, and confirmation of their acceptance of the appointment.<br>¬ In the initial round, experts received an explanatory invitation letter, along with the data collection schedule, informed consent document, and data collection questionnaire, with a deadline of 7 days for their response.<br>¬ After seven days, the researcher provided the first-round results to the experts with the collected data, consolidated judgments, respective comments, and descriptive statistics related to the obtained scores. This process continued iteratively until a consensus was reached.<br>¬ Experts who failed to provide feedback within the stipulated 7-day timeframe were excluded from subsequent stages.

									¬ Two quantitative measures were utilised to determine expert consensus on item inclusion: the Percentage Score Calculation and the Content Validity Index (CVI) Calculation. For percentage score calculation, the cut-off point was set at questions (statements) receiving 90% total agreement (score 5) and partial agreement (score 4). The CVI calculation utilised a cut-off point exceeding 78%.
Licen and Plazar (2019) [70]	Journal of Nursing Scholarship	Developing a universal nursing competencies framework for registered nurses: A mixed-methods approach	Slovenia	Develop a methodology to identify a set of universal nursing competencies that contribute to effective nursing performance in Slovenia.	14	14	0,0%	4	¬ The authors conducted a literature review on the research subject to compile a predefined list of items for the Delphi survey. However, they opted to initiate the first round exclusively with an open-ended question, deferring the introduction of literature review results to the second round.<br>¬ The selection criteria for experts recruited for the Delphi panel included: 1) recognition for their exceptional work in Slovenian nursing; 2) availability to participate throughout all four rounds of the research project; 3) commitment to communication via email to ensure anonymity among the panellists.<br>¬ An introductory email and a letter of invitation were sent to 28 potential participants, out of which 17 responded affirmatively. Ultimately, 14 experts participated in the study.<br>¬ The first round commenced with an open-ended question, and responses were systematically organised in a spreadsheet based on semantic similarity.<br>¬ In the second round, items identified in the literature review were introduced, and panellists were invited to review the spreadsheet and suggest additional nursing competencies. Additionally, this round allowed panellists to provide comments next to each competency.<br>¬ Subsequently, in the third round, the refined nursing competencies were presented for evaluation using a 3-point Likert scale (ranging from 1 [disagree] to 3 [agree]). The fourth round involved experts evaluating the selected nursing competencies using a 5-point Likert scale (ranging from 1 [not at all important] to 5 [very important]). No new competencies could be added at this stage.<br>¬ Panel opinions were measured using averages, while standard deviations were utilised to represent the level of consensus within the panel.<br>¬ Consensus in the final two rounds was defined as the general agreement of at least 80% of the panel members.
Beauvais and Phillips (2020) [59]	Nursing Education Perspectives	Incorporating future of nursing competencies into a clinical and simulation assessment tool: Validating the clinical simulation	United States of America	The study aimed to assess the content validity of the Clinical Simulation Competency Assessment Tool (ClinSimCAT).	42	16	-61,9%	3	¬ Establishment of a task force for literature review and proposition of an initial set of competencies. The study participants were recruited through convenience and snowball sampling. They were contacted via email using lists of deans of nursing schools. Additionally, the authors searched nursing school websites for nursing faculty, directors of nursing undergraduate

		competency assessment tool						programs, simulation centres, and nurse educators with specialised knowledge relevant to the project.<br>¬ In the first round of the Delphi survey, participants were asked to respond to demographic questions and evaluate each competency's relevance, specificity, and comprehensibility using a 5-point Likert scale (1 = not at all, 3 = somewhat, and 5 = completely).<br>¬ In rounds 2 and 3 of the Delphi survey, participants were requested to assess each competency with adjustments based on the previous round's feedback.<br>¬ Responses rated as 4 or 5 were considered as indicating competency.
He, Zhou, Zeng and Ma (2021) [60]	Nurse Education Today	Development of the competency assessment scale for clinical nursing teachers: Results of a Delphi study and validation	China	Define the elements for assessing the competencies of clinical nursing supervisors and test the reliability and validity of the scale.	40	40	0,0%	3	¬ The competencies defining clinical nursing supervisors were identified through a literature review. Subsequently, these elements were discussed and organised to form the initial version of the measurement tool.<br>¬ Criteria for expert selection were established: 1) a minimum of 10 years of experience in clinical practice, nursing education, and nursing management; 2) holding at least a bachelor's degree with a senior position; and 3) voluntary participation and commitment until the conclusion of the expert consultation process.<br>¬ Members of the research team personally contacted Experts to clarify the research objectives and obtain their consent.<br>¬ The first consultation round was conducted in person, as a group meeting, where questionnaires were distributed and individually completed.<br>¬ Subsequent rounds involved distributing questionnaires via email, with experts anonymised from each other's feedback. Feedback was collected via email.<br>¬ Continuous data were presented as means and standard deviations, while categorical data were shown as absolute frequencies (n.) and relative frequencies (%). Coordination and concentration of expert opinions were assessed using Kendall's W and coefficient of variation (CV). The expert authority coefficient (Cr) was also calculated.<br>¬ Consultations were considered complete when the approval rate from experts and the inclusion rate of items in the competency assessment tool for clinical nursing supervisors exceeded 92.5%. Items were included if their mean importance score was above 4 points and had a coefficient of variation below 0.25 (indicating consensus).
Janssens, Van Hauwe, Ceulemans and Allegaert (2021) [61]	International Journal of Environmental Research and Public Health	Development and pilot use of a questionnaire to assess the knowledge of midwives and pediatric nurses on maternal use	Belgium	To develop and test a questionnaire on obstetric nurses' and paediatric nurses' knowledge of	7	3	-42,8%	2	¬ Before the Delphi panel convened, a literature review was conducted to establish a set of elements categorised into five subgroups.<br>¬ Information derived from the literature review was further refined through two focus groups. These aimed to identify pertinent themes and items for

		of analgesics during lactation		analgesics (acetaminophen, ibuprofen, aspirin, tramadol, codeine, oxycodone) during lactation, using a structured, stepwise approach.					inclusion in the initial questionnaire: one group consisted of obstetric nurses (4 participants), while the other comprised pediatric nurses (6 participants).<br>¬ In the first round, experts were selected to encompass diverse expertise in content areas (e.g., nursing, medicine, pharmacology) and methodological perspectives, including experience in questionnaire development and measurement instruments (7 experts).<br>¬ For the second round, the number of experts was determined through a selection process among those initially included. The criterion for selection was the absence of "atypical" evaluations in the first round, involving low or high scores compared to the evaluations of other experts, combined with a qualitative assessment of individual feedback on items.<br>¬ During the first round, content validity was assessed individually by all experts. In the second round, the retained items were reassessed for content validity by experts from the initial group.<br>¬ Each item was scored on a 4-point Likert scale (1 = not relevant; 2 = partially relevant; 3 = relevant; 4 = very relevant).<br>¬ The content validity index (CVI) was calculated for each item in the first round. In the second round, the content validity index for each item and the scale's overall content validity index were calculated.<br>¬ Items with a CVI < 0.78 were eliminated.
Zhang, Zhou, Wang, Luo and Li (2021) [62]	Asian Nursing Research	Development and validation of the humanistic practice ability of nursing scale	China	Developing a Humanistic Nursing Practice Ability (HPAN) scale adapted to China and testing its psychometric properties.	16	16	0,0%	2	¬ Five primary indicator competencies were established based on the HPAN conceptual framework and a literature review, utilising the original HPAN evaluation index as a foundation.<br>¬ Sixteen experts holding senior professional titles and engaged in humanistic nursing research were recruited for the expert panel.<br>¬ Theoretical and operational definitions of the scale concept were provided to each expert. They were asked to assess the clarity and relevance of each item using a 4-point scale (4 = "very pertinent", 3 = "pertinent but requires minor revision", 2 = "pertinence cannot be assessed without reviewing the item", 1 = "not pertinent"). Comments were solicited for each item. The content validity index (CVI) for each item was calculated based on the proportion of experts rating it as 3 or 4. Items with a CVI lower than 0.80, typically indicating high validity, were eliminated after review. Concurrently, experts were consulted regarding the semantics and accuracy of scale items. When consensus was reached among experts, the consultation concluded.<br>¬ The authority coefficient of the experts was determined based on their academic level, judgment criteria, and familiarity with the scale. In this study, the authority coefficient of the experts was 0.85.

Mei, Chang, Zhu, Dong, Zhang and Zeng (2022) [71]	Nursing Open	Core competency scale for operating room nurses in China: Scale development, reliability and validity evaluation	China	To develop a competence scale for operating theatre nurses in China and provide evidence of the scale's reliability and validity.	10	10	0,0%	2	¬ The competency requirements (domains of the scale) for perioperative nurses were identified through two focus groups, where ten perioperative nurses were conveniently recruited for interviews.<br>¬ Before consulting the Delphi panel experts, the research team and five external experts from the Nursing Association thoroughly discussed the categories of domains identified in the focus group interviews, along with the associated items for each domain, to reach a consensus. The initial version of essential competencies for perioperative nurses was then developed based on the focus group results and extensive discussions. This version was subsequently distributed to panel members for their feedback.<br>¬ The panel consisted of experts with over ten years of professional experience in operating theatre nursing practice, nursing education and training, and nursing management. Qualified experts were selected from the senior members of a nursing association and invited to join the panel. Ten experts provided informed consent and participated as panellists for the expert consultation.<br>¬ The outcomes of the expert consultation process were evaluated using the experts' positive coefficient, degree of authority, and coordination coefficient.
Penataro-Pintado, Rodriguez-Higueras, Llaurado-Serra, Gomez-Delgado, Llorens-Ortega and Diaz-Agea (2022) [63]	International Journal of Environmental Research and Public Health	Development and validation of a questionnaire on the perioperative nursing competencies in patient safety	Spain	To develop and validate the CUCEQS©, an instrument for measuring perioperative nurses' perceived competence in relation to surgical patient safety.	38	37	-2,6%	2	¬ An extensive literature review focused on structures and competency models defined by organisations with scientific authority in the studied topic.<br>¬ Five focus groups were organised to discuss critical aspects of surgical patient safety.<br>¬ Based on the analysis of these focus groups, the research team developed the CUCEQS© questionnaire. Reference recommendations from organisations with scientific authority on the subject and national and international strategic lines on patient safety were used to define the instrument, its components (competencies, sub-competencies, and items), and its structure.<br>¬ Experts were selected based on specific criteria:<br>¬ Perioperative nurses: 1) with over five years of continuous professional experience, predominantly full-time, in operating theatres or postoperative units of second or third-level public hospitals in Spain; 2) currently employed in such roles; and 3) possessing a minimum of specific postgraduate training in perioperative nursing.<br>¬ Clinical safety experts: 1) members of the institution or association boards of directors or safety committees in their workplace, with extensive public and scientific background in patient safety.

									¬ Perioperative nurse experts were contacted through their supervisors, while clinical safety experts were reached via email using provided or publicly available contact information.<br>¬ The panel of experts was intentionally selected, with 40 potential experts in Spain initially contacted; 38 agreed to participate, and 37 completed both rounds.<br>¬ In this study, the focus group stage was considered equivalent to one round of the Delphi panel. Each round had a 4-week response period, with multiple reminders sent to maximise responses. The entire process took three months to complete.<br>¬ Data analysis involved examining the means and standard deviations of each item and sub-competency of the questionnaire. Adequate results were considered to have a mean score > 4 out of 5 and a Content Validity Index (CVI) > 0.78.<br>¬ In both rounds, suggestions were made regarding the distribution and improvement of sub-competencies and items' wording.
Wang, Tong, Wang and Zhang (2022) [64]	International Journal of Environmental Research and Public Health	A study on the nurse manager competency model of tertiary general hospitals in China	China	To build a scientific and practical model of nurse manager competencies that hospitals can use as a tool to select nurse managers.	20	19	-5,0%	2	¬ The study commenced by identifying the competencies of nurse managers through a literature review and interviews, integrating all obtained competencies as preliminary for subsequent selection and model definition.<br>¬ Experts were selected at the national level based on educational qualifications, professional titles, professional experience, training, and positions held. All experts possessed over five years of professional experience in nursing management.<br>¬ Results indicated that the coefficient of the degree of authority for all factors in both rounds exceeded 0.8, indicating high reliability in the experts' assessments. Additionally, Kendall's coordination coefficient for all factors was statistically significant ($p < 0.05$), suggesting high validity of the results from both rounds.

INE – Initial number of experts; FNE – Final number of experts; EDOR – Expert drop-off rate; TNR – Total number of rounds.
